# Assessment of the impact of manual curation in BioCyc

**DOI:** 10.3389/fbinf.2026.1887950

**Published:** 2026-08-27

**Authors:** R. Caspi, B. Wilson-Mortier, L. R. Moore, J. N. Karp, D. Beesley, I. T. Paulsen, P. D. Karp

**Affiliations:** 1 Koonkie Inc., Menlo Park, CA, United States; 2 Biomolecular Discovery Research Centre, Macquarie University, Sydney, NSW, Australia; 3 Bioinformatics Research Group, Artificial Intelligence Center, SRI, Menlo Park, CA, United States; 4 Williams College, Williamstown, MA, United States

**Keywords:** annotation errors, curation, genome annotation, genome annotation errors, genome database

## Abstract

**Introduction:**

BioCyc is an extensive collection of databases of genomic and pathway information for microorganisms and model eukaryotes. These organismal databases integrate diverse biological data by combining computationally inferred information, data imported from other databases, and, for selected organisms, literature-based manual curation. This study investigates the magnitude and significance of annotation changes performed during the curation of 10 prokaryotic genomes to better understand the rate of erroneous annotations and the value of BioCyc curation.

**Methods:**

We identified curation changes by finding cases where the annotation of the protein at the start of the curation process differed from its annotation at the end of the process.

**Results:**

We found that across a sample of curated databases (*n* = 10), the annotation of 6,753, or 25.6% of the proteins in the pooled protein dataset (n = 26,126) were modified. Assessment of considerable sampling fractions of these proteins found that a median of 62% (mean of 52.9%) represented functionally informative name changes, rather than stylistic annotation changes. These results were then extrapolated to total proteins with name changes with uncertainty quantified via finite population correction, indicating that most Tier 2 Biocyc PGDBs received hundreds of functionally informative name changes during manual curation. On average 363, or13% (±5.4% SD) of the proteins encoded in each genome received functionally informative annotation changes, ranging from 5.3% *(Streptococcus pneumoniae* D39V) to 22.7% (*Staphylococcus aureus* NCTC 8325).

**Discussion:**

These findings demonstrate a substantial improvement in the accuracy of manually curated BioCyc databases compared with automated annotation pipelines. This result is particularly impactful as the rate of downstream propagation of erroneous annotations across biological databases can significantly compromise scientific discovery.

## 1 Introduction^1^

High-throughput sequencing technologies have yielded more than 100,000 bacterial genomes. This large number of genomes has necessitated reliance on automated genome annotation pipelines such as NCBI’s Prokaryotic Genome Annotation Pipeline (PGAP), Prokka, or Bakta (Seemann, 2014; Tatusova et al., 2016; Schwengers et al., 2021). Despite their utility, outside of specialist circles it is often underappreciated that automated genome annotation systems are prone to misannotations, that is, to assigning an incorrect functional annotation to a protein.

Misannotated genes can lead to errors in pathway reconstruction, flawed metabolic models, wasted reagents, wasted instrument time, and wasted personnel time. For example, imagine that a gene-expression experiment identifies several genes as important in a particular phenotype, but one is misannotated. Considerable time could be lost based on incorrect assumptions about its function. Strain engineering companies could pursue doomed engineering strategies based on misannotations. Drug discovery companies could pursue drug targets with incorrect functions in efforts that are also ill-fated. Ultimately, misannotations can compromise scientific results and give rise to erroneous publications and grant proposals. The annotation process also tends to propagate misannotations between scientific databases. For example, Riccardo Percudani from the University of Parma in Italy discovered that a group of enzymes (ureidoglycolate lyase, which releases urea) had been inaccurately assigned the function of ureidoglycolate hydrolase (which releases ammonia) in almost all databases, and the error had infiltrated numerous research projects (Percudani et al., 2013). Percudani commended MetaCyc (which is a BioCyc database containing experimentally studied pathways and enzymes from all domains of life) as the sole database with accurate information about this enzyme, due to the manual curation process integral to MetaCyc (Caspi et al., 2020). Although several studies of the accuracy of genome annotation systems have been performed, we still lack a precise and universally applicable solution to this problem. False annotations fall into two classes: false-positives (an incorrect annotation asserted for a protein for which no function can properly be inferred) and false-negatives (where an incorrect or no functional annotation is inferred for a protein for which a function can be properly inferred).

Brenner (1999) showed that three different annotations of the *Mycoplasma genitalium* genome showed an 8% rate of disagreement (this method cannot detect cases where all methods agree on an incorrect annotation). Devos and Valencia (2001) estimated that as many as 30% of genome annotations may be incorrect. Radivojac et al. (2013) showed that BLAST-based annotation achieved an F-measure of 0.4. F-measure is a harmonic mean between precision and recall that ranges from 0 (precision or recall are 0) and 1 (precision and recall are both perfect–no false positives), whereas newer annotation methods achieved an F-measure of 0.6 (Jiang et al., 2016).

This article is concerned with those subsets of proteins with false annotations that have undergone substantial published experimental characterization and are therefore amenable to correction via literature-based database curation methods (Howe et al., 2008). In the remainder of the article, we outline the curation strategy used by the BioCyc project and estimate the number of annotation errors corrected by BioCyc curators.

## 2 Database annotation in BioCyc

The BioCyc.org genome and metabolic pathway web portal creates rich and detailed portraits of sequenced organisms by combining computational inferences, data imports, and manual curation. Each BioCyc database describes one sequenced organism through an extensive object-oriented database schema whose classes define the genome, proteome, reactome, metabolome, metabolic pathways, and regulatory network of the organism. These databases enable fuller understanding of gene functions by embedding genes in their metabolic pathway(s) and regulatory contexts. BioCyc provides an evidence-based foundation of information distilled from more than 167,000 publications that enable more accurate hypothesis formation and improved interpretation of experiments.

BioCyc database creation begins with annotated genomes provided by NCBI RefSeq, as they provide standardized annotations across thousands of genomes. Our extensive bioinformatic pipeline supplements RefSeq-derived annotations with:

- Predicted transport reactions, metabolic reactions, and metabolic pathways
- Inferred operons
- Inferred protein complexes
- Candidate genes encoding missing metabolic enzymes (pathway hole fillers)
- Cross-genome protein ortholog group assignments

**FIGURE 1:**
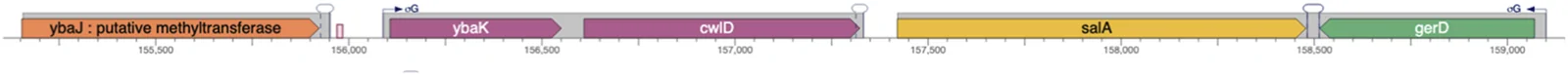
Image from the BioCyc Genome Explorer genome browser from *B*acillus*. subtilis* 168 showing how imported genome information (e.g., transcription start sites illustrated by arrows at the beginning of operons and termination sites illustrated as stem-loops at the ends of genes) and predicted genome information (such as operon predictions, illustrated as adjacent genes in the same color) are shown together.

Data import tools integrate information from other bioinformatics databases and publications. Examples include importing Gene Ontology annotations and protein feature data from UniProt (Uniprot Consortium, 2025), and in some cases, gene essentiality datasets, transcription start sites, ribosome binding sites, and terminator sites (see Figure 1). However, up to this point in the process the databases are based on the original protein functions.

**FIGURE 2:**
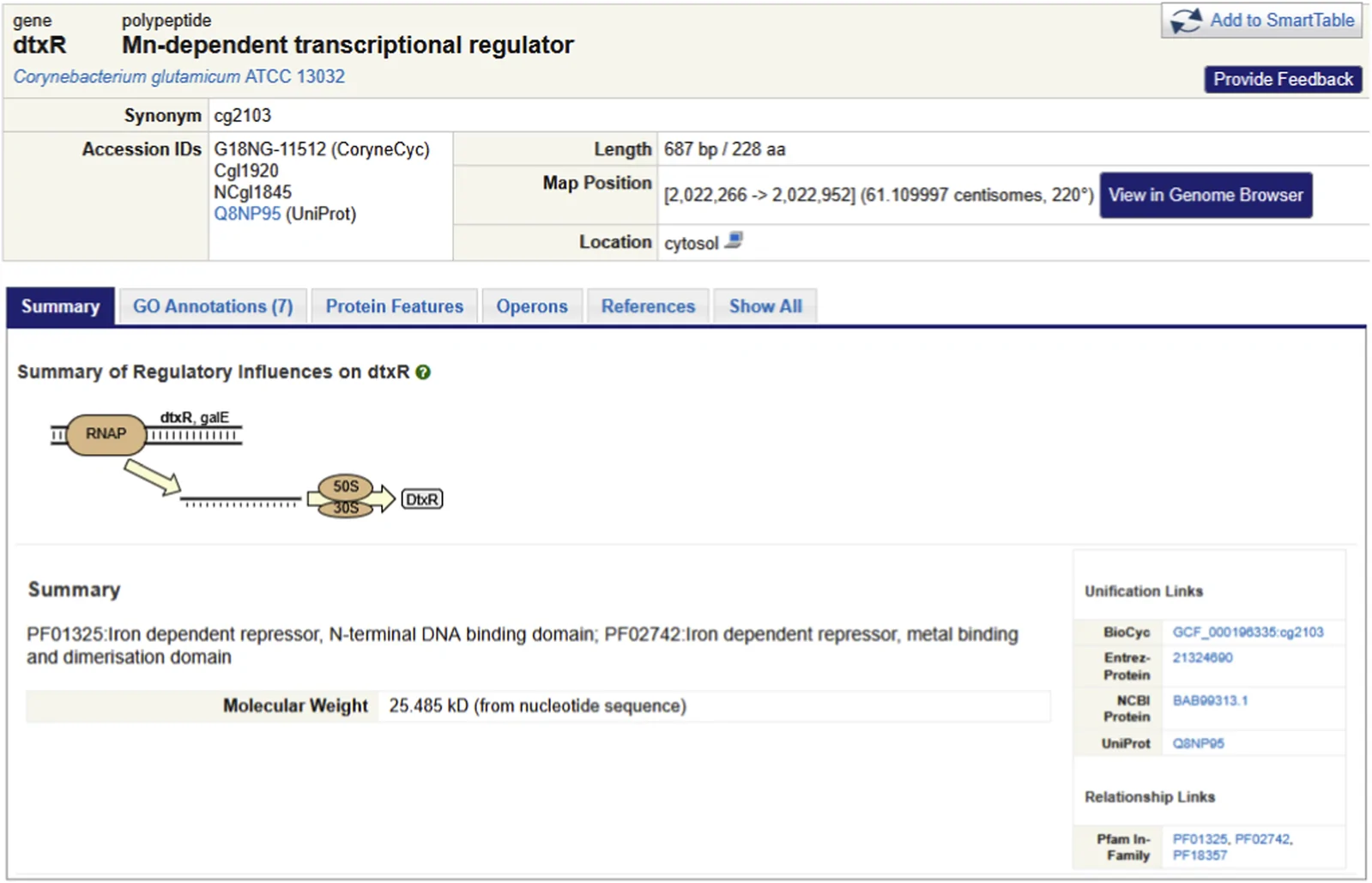
The protein page for the product of the *dtxR* gene of *Corynebacterium glutamicum* ATCC 13032 prior to manual curation.

**FIGURE 3:**
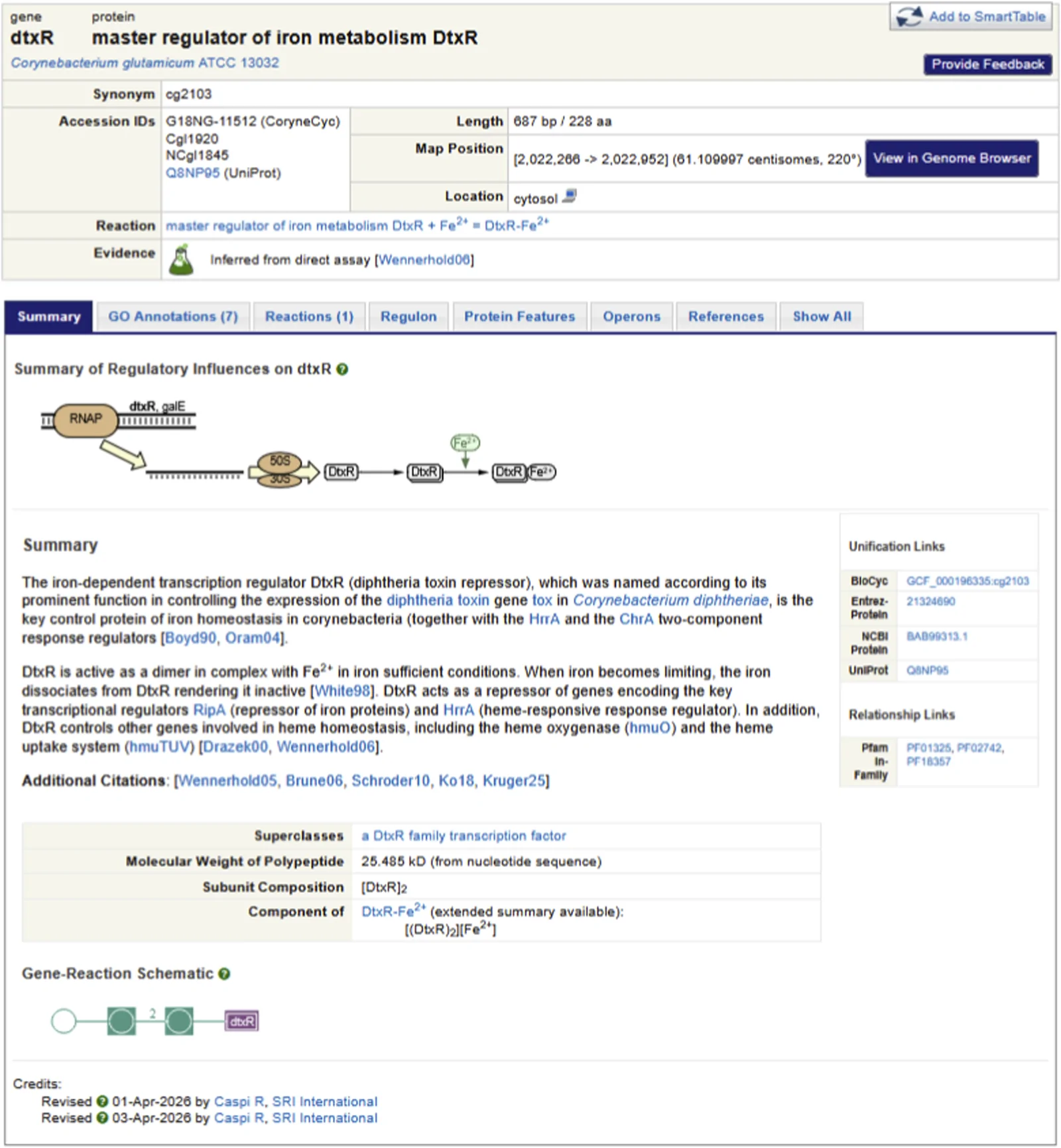
The protein page for the product of the *dtxR* gene of *Corynebacterium glutamicum* ATCC 13032, following manual curation. Changes made during curation include name change, the creation of a dimeric complex, definition of the interaction with Fe^2+^, and composition of a short summary, including multiple references to the original literature. In addition, an experimental code was added and the binding sites of the regulator to its targets were defined (the regulon is displayed in a different tab, see Figure 4).

**FIGURE 4:**
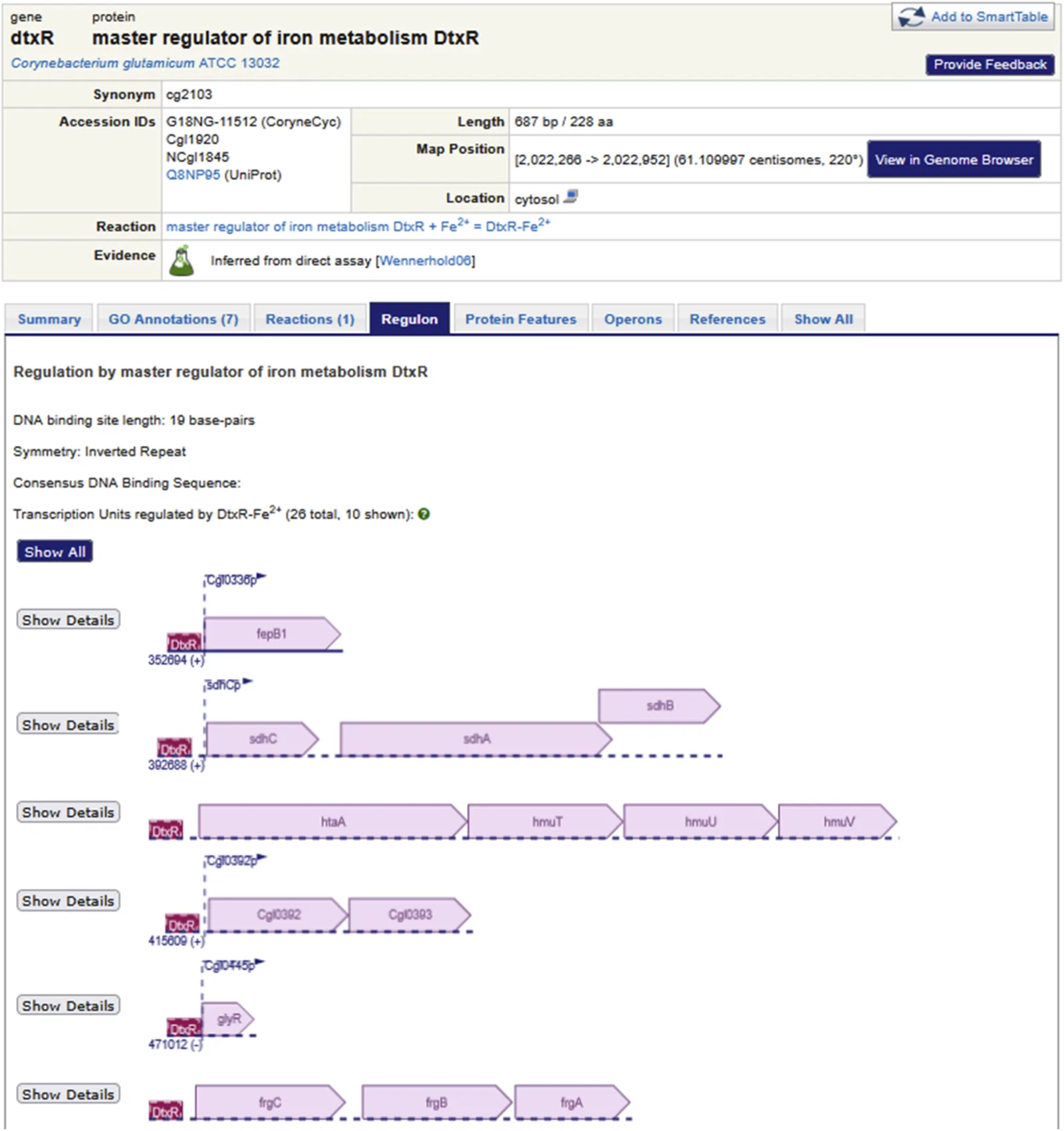
The regulon of DtxR of *Corynebacterium glutamicum* ATCC 13032. To conserve space, only a subset of the regulated genes is shown in this image. The regulatory information here was curated manually. The binding site data was taken from the RegPrecise database (Novichkov et al., 2013).

To combat the problem of incorrectly annotated proteins, BioCyc applies extensive literature-based manual curation to improve the quality and accuracy of selected high-priority databases. These updates include, but are not limited to, the addition, removal, or modification of database objects such as gene and protein annotations, enzymatic reaction assignments, and predicted metabolic pathways. Curators also author mini reviews up to several paragraphs in length for genes and pathways that contain extensive references to experimental literature, thus providing a paper trail that justifies the annotation changes. In addition, curators assign evidence codes that identify protein functions as determined experimentally, a practice pioneered by Junker et al. (1999). Examples for evidence codes are “assay of protein purified to homogeneity from a heterologous host” or “reaction blocked in mutant”. Each evidence code is backed by a citation to the original literature where the function was demonstrated. Examples of a curated protein are provided in Figures 2–4.

The changes in protein functionality that are uncovered during manual curation often lead to changes in the metabolic pathways, as some of the originally predicted enzymatic activities no longer exist in the database, while new activities are added. The curators carefully reassess the metabolic pathways, deleting those that were originally predicted but are no longer supported, and adding new pathways that incorporate the new activities.

The gold standard of evidence required for functional annotation changes in BioCyc is therefore organism-appropriate published experimental data. In some cases, curators may rely on strong computational evidence to infer the functions of misannotated proteins (e.g., when an ortholog has been experimentally characterized from a closely related species). However, such cases require informed judgement on a case-by-case basis and are accompanied by an appropriate evidence code.

We refer to databases that have undergone one person-year or more of curation as Tier 1 databases; databases that received more than one person-month but less than a year as Tier 2 databases, and databases that received no manual curation as Tier 3 databases. Since Tier 3 databases received no manual curation, they contain the annotation errors present in the original annotated genomes.

## 3 Methods

Phase I. To better understand the extent by which curation improves the databases, we conducted a manual review of 1,907 proteins whose names were changed during curation, gathered from 10 Tier 2 BioCyc databases for bacterial genomes. The 10 databases, which are listed in Table 1, were all curated in the last 5 years. Our study focuses on Tier 2 databases created in the last 5–10 years because our Tier 1 databases (and some Tier 2 databases) are significantly older than this. For technical reasons we do not have the database update histories of these older PGDBs, which are required for us to identify protein name changes. These 10 databases are representative of the larger collection of curated BioCyc databases in that they cover a range of genome sizes, and a range of taxonomic groups, and have undergone similar amounts of curation.

**TABLE 1:** Phase I analysis of manual curation changes made to 10 BioCyc databases.

Organism database	A total protein- coding genes	B total proteins with name changes	C percent proteins modified (B/A)	D phase I sample size	E sampling fraction (D/B)	F number functional	G number stylistic	H proportion functional (F/D)
*Acinetobacter* baumannii ATCC 17978	3636	733	20.2	200	0.273	101	99	0.505
*Chlamydia trachomatis* D/UW-3/CX	889	110	12.4	108	0.982	67	41	0.620
*Enterococcus faecalis* V583	3193	596	18.7	200	0.336	124	76	0.620
*Helicobacter pylori* 26695	1445	524	36.3	200	0.382	53	147	0.265
Lactiplantibacillus plantarum WCFS1	3014	960	31.9	199	0.207	126	73	0.633
*Neisseria gonorrhoeae* FA 1090	1934	354	18.3	200	0.565	131	69	0.655
*Staphylococcus aureus* NCTC 8325	2825	1020	36.1	200	0.196	126	74	0.630
*Streptococcus* pneumoniae D39V	1991	608	30.5	200	0.329	35	165	0.175
Synechocystis sp. PCC 6803	3630	881	24.3	200	0.227	131	69	0.655
*Vibrio cholerae* O1 biovar El Tor str. N16961	3569	967	27.1	200	0.207	107	93	0.535
Sum or Average	26126*	6753*	25.6 ± 8.1**	190.7 ± 29.1**	0.370 ± 0.242**	100.1 ± 35.6**	90.6 ± 38**	0.529 ± 0.172**

* Value represents a sum.

** Value represents a mean (±SD).

To generate the initial randomized phase I samples, protein annotations were compared before and after curation for each PGDB via string matching (case-insensitive). The lists were randomized, and curators assessed ∼200 proteins from each list (note: *Chlamydia trachomatis* D/UW-3/CX and *Lactiplantibacillus plantarum* had 108 and 199 annotations assessed, respectively). Curators were then assigned with the task of identifying whether protein name changes represented a change in the functional annotation of the protein (a *functionally informative change*), or merely a stylistic annotation change (*stylistic change*).

Stylistic changes in BioCyc ensure consistency with established naming conventions for proteins. Examples include changing a protein originally annotated as “APC family permease” to “putative transporter, APC family”, or modifying an enzyme name to match the annotation assigned by the Enzyme Commission (EC). Adherence to standardized naming conventions is vital as nomenclature standardization remains a significant issue in biology, partly due to the great number of annotation methods available. This effort ensures consistency both within and across PGDBs and is subsequently indispensable when performing comparative analyses.

The changes in functional annotations for BioCyc proteins fell within four categories. The categories, listed below, covered new functional annotations for (1) proteins previously annotated as ‘hypothetical protein’, (2) complete re-assignments in proposed functions, and (3/4) functional clarifications correcting overly precise or imprecise annotations:

- No function > Some Function (“hypothetical protein” > “peptide pheromone SHP1518”)
- Function A > Function B (“laminin-binding surface protein” > “Zn^2+^-binding lipoprotein”)
- General Function > Specific Function (“glycosyltransferase family 25 protein” > “UDP-N-acetyl-galactosamine:lipooligosaccharide-beta-1,3-N-acetylgalactosaminyltransferase LgtD”)
- Specific Function > General Function (“malonate permease” > “putative transporter”)

Phase II. We then performed a more thorough analysis of total curation changes in each PGDB from phase I. During this phase we extracted and quantified:

- Task 1: Changes in protein names across each genome
- Task 2: Changes in reaction assignments for metabolic enzymes
- Task 3: Changes in the presence or absence of metabolic pathways

Task 2 identified changes in enzyme functionality by comparing reaction assignment before and after the curation process. Task 3 expanded the data analysis to the curation of metabolic pathways. Changes to both reaction assignments for metabolic enzymes and pathways reflect experimental data in addition to the removal of incorrect pathway predictions, such as pathways for the wrong quinone or the wrong number of isoprenoid subunits. As explained above, changes in enzyme functionality often lead to changes in the metabolic network, and part of the manual curation process comprises deletion of pathways that are no longer supported and the introduction of new pathways that are associated with the new activities. Task 3 therefore identified pathways that were either deleted or introduced during curation.

Given the non-negligible sampling fractions of the data assessed during phase I, we then estimated the total number of curated proteins with significant name changes in each PGDB. To achieve this, phase I proportions for each genome were extrapolated to the full set of proteins with name changes in each genome (using data from Task 1 above, but excluding Tasks 2 and 3). To quantify uncertainty around these estimations, approximate 95% confidence intervals (CI) were calculated for the proportions (assuming they are Bernoulli random variables) using finite population correction. Relative CI half-widths were used to assess precision of the estimates, with a practical threshold of 10% used as a benchmark to flag potential, but not concrete, cases of lower precision.

## 4 Results

Phase I Results. In the phase I survey, we found that the percentage of name changes ranged from 12.4% (*Chlamydia trachomatis* D/UW-3/CX) to 36.3% (*H. pylori* 26695), with an average of 25.6% (±8.1% SD) across all PGDBs (Table 1, column C). Assessment of the phase I subsets (*n* = ∼200) found that on average, approximately half of the updates (52.9% ± 17.2% SD) represented functionally informative changes rather than stylistic changes (Table 1, column G). Despite the large variance, the median proportion of functional name changes was 0.620, with an interquartile range of 0.513–0.632, and a total range of 0.175–0.655. Only two PGDBs (*H*. *pylori* 26695 and *S. pneumoniae* D39V) displayed proportions below the lower Tukey fence (0.333-0.812).

Although *S*. *pneumoniae* D39V displayed the lowest proportion of functionally informative changes across all PGDBs, this was not unexpected, as the assembly was pre-curated by the Veening lab (Slager et al., 2018; Janssen et al., 2025). Similarly, the genome of *H. pylori* 26695 was first sequenced in 1997 and had undergone substantial experimental characterization and manual curation in dedicated databases such as PyloriGene before the introduction of NCBI’s PGAP pipeline (Tomb et al., 1997; Boneca et al., 2003). As annotation algorithms are based on homology transfer, well-studied model organisms are more likely to contain higher-quality annotations than those from divergent lineages.

Sampling fractions of the phase I subsets, calculated as phase I sample sizes divided by total proteins with name changes in each PGDB, were non-negligible (Table 1, column E). These ranged from 0.196 for *S*. *aureus* NCTC 8325, to 0.336 for *Enterococcus faecalis* V583, but included one significant outlier (0.982 for *C. trachomatis* D/UW-3/CX). The phase I sample size of the *C. trachomatis* was much smaller than the rest (*n =* 108). This organism possesses a much smaller genome than the other dataset organisms (889 protein-coding genes, 110 of which had name changes). Therefore, the phase I subset of this organism reached almost 100% coverage. With the exclusion of this genome, sampling fractions displayed an average of 0.302 (±0.119 SD).

**FIGURE 5:**
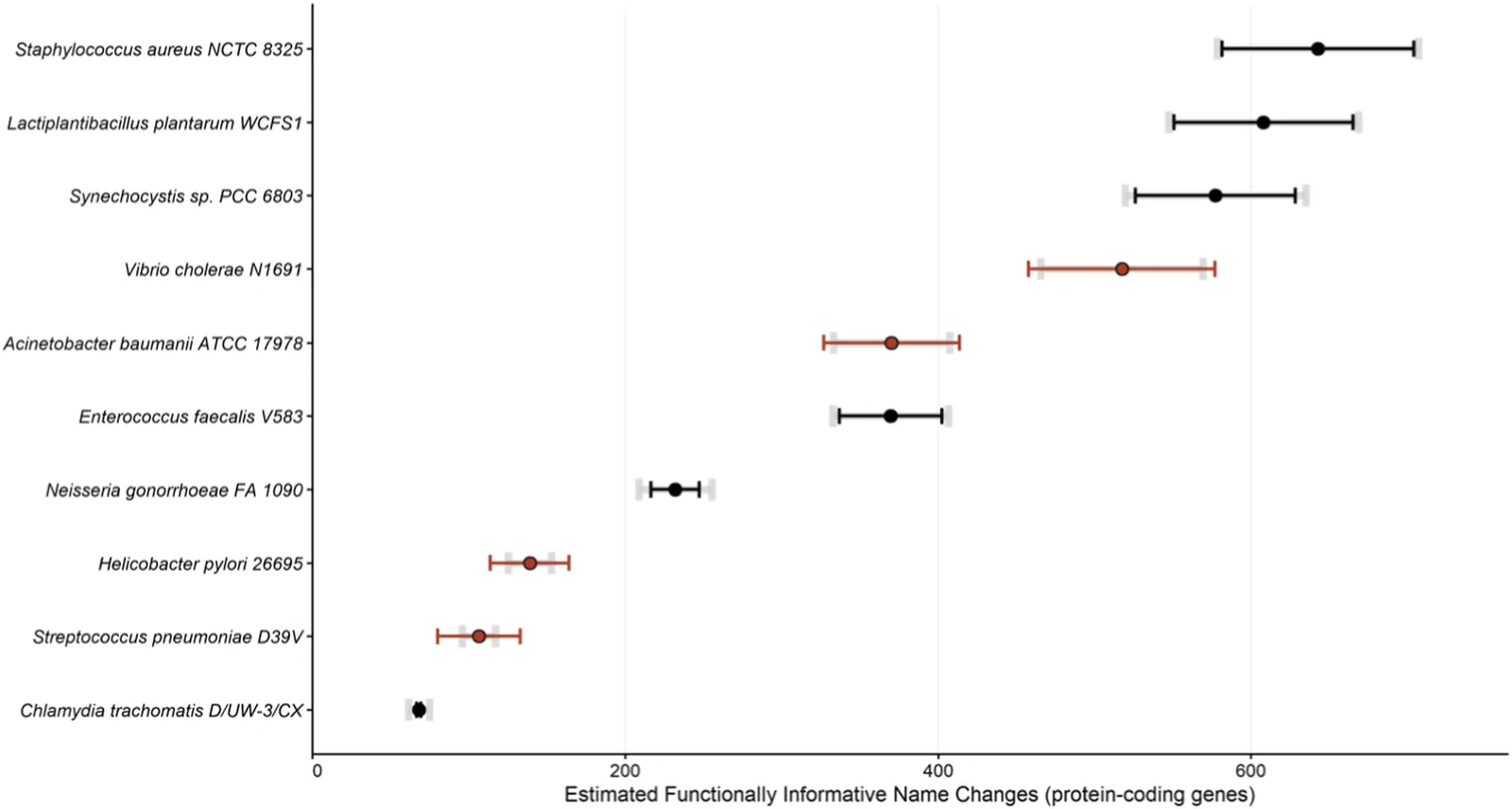
Estimated number of functionally informative name changes for each manually curated PGDB. Estimates were obtained by extrapolating the observed proportion of functionally significant name changes in the assessed subset to the full set of proteins with name changes in each PGDB. Error bars represent 95% confidence intervals calculated using finite population correction. Phantom bars represent the relative CI half-width chosen to assess precision (10%), Red intervals exceeded these ranges.

Phase II Results. Across all 10 genomes in the analysis, BioCyc curation resulted in the modification of a total of 6,753/26,126 protein names, representing updates to 25.8% of the aggregated proteomes (Table 2). With the exclusion of *C. trachomatis*, estimations of functionally informative changes ranged from 138.9 for *H. pylori* 26695 to 642.6 for *S. aureus* NCTC 8325 (Figure 5). All but four CI half-widths fell below the chosen cutoff (10%), but only *H. pylori* 26695 (18.1%) and *S. pneumoniae* D39V (24.7%) deviated significantly, owing to their lower phase I proportions. Therefore, under the assumption that the phase I subsets were representative of the total populations of proteins with name changes in each PGDB, the estimates can confidently be estimated to lie within the calculated ranges.

**TABLE 2:** Phase II analysis of enzymes and pathways. For convenience, Column A and B replicate Columns A and B from Table 1.

Organism database	A total protein- coding genes	B proteins with name changes	C estimated proteins with function changes ( ± CI)***	D total metabolic enzymes	E enzymes with altered reactions	F percent of enzymes altered	G total pathways before curation	H pathways added or removed	I total curation changes
*Acinetobacter baumannii* ATCC 17978	3636	733	370.2 ± 43.3	1083	468	43.2	242	63	1264
*Chlamydia trachomatis* D/UW-3/CX	889	110	68.2 ± 1.4	297	74	24.9	80	20	204
*Enterococcus faecalis* V583	3193	596	369.5 ± 32.7	884	397	44.9	237	109	1102
*Helicobacter pylori* 26695	1445	524	138.9 ± 25.2	666	379	56.9	162	79	982
*Lactiplantibacillus plantarum* WCFS1	3014	960	607.8 ± 57.2	973	558	57.3	206	143	1661
*Neisseria gonorrhoeae* FA 1090	1934	354	231.9 ± 15.4	766	306	39.9	188	76	736
*Staphylococcus aureus* NCTC 8325	2825	1020	642.6 ± 61.2	850	441	51.9	153	91	1552
*Streptococcus pneumoniae* D39V	1991	608	106.4 ± 26.2	769	316	41.1	236	107	1031
*Synechocystis* sp. PCC 6803	3630	881	577.1 ± 51	1113	641	57.6	244	125	1647
*Vibrio cholerae* O1 biovar El Tor str. N16961	3569	967	517.3 ± 59.5	1221	502	41.1	303	84	1553
Sum/Average^*^	26126*	6753*	363 ± 218.1**	8622*	4082*	45.9 ± 10.3**	2051*	89.7 ± 32.7**	1173.2 ± 441.7**

* Value represents a sum.

** Value represents a mean (±SD).

*** Column values (excluding mean and standard deviation) include estimation ± finite population corrected 95% confidence intervals.

Focusing on a single organism, the genome of *L*. *plantarum* WCFS1, an industrially significant and highly characterized lactic acid bacterium (LAB), received 960 updated protein annotations (31.9% of total proteins with name changes), of which 607.8 (±57.2 CI) were estimated to be functionally informative changes (Table 2; column C). Full statistics regarding curated changes regarding enzymes, pathways, and reactions for PGDBs can also be seen in Table 2. For *L. plantarum*, curators also made 69 pathway additions and 74 removals (143 total pathway modifications, or 69.4% of predicted pathways present before manual curation); and 558 modifications to metabolic enzyme reactions (57.3% of all metabolic enzymes). In total, 1,661 modifications were made to this database (Table 2, column I), calculated as the sum of modified protein annotations, enzyme reaction assignments, and pathways added or removed. Examples of annotation changes made to *L. plantarum* WCFS1, both functional and stylistic (e.g., change in name format), can be seen in Table 3.

**TABLE 3:** Examples of protein annotation updates in the genome of *L. plantarum* WCFS1.

Accession	Initial function	Revised function	Type of change	Justification
lp_1229	MucBP domain-containing protein	Mannose-specific adhesin	Assignment of function	Function described in literature (Pretzer et al., 2005; Gross et al., 2010)
lp_1204	EpsG family protein	Capsular polysaccharide 2 polymerase Cps2H	Assignment of function	Function described in literature (Remus et al., 2012)
lp_0796	Alpha/beta fold hydrolase	Hydroxycinnamic acid esterase	Assignment of function	Function described in literature (Esteban-Torres et al., 2013; Navarro-González et al., 2013)
lp_0185	PTS beta-glucoside transporter subunit IIBCA	Sucrose-specific PTS 1 enzyme IIBCA	Change of function	Function described in literature (Saulnier et al., 2007)
lp_3479	LacI family DNA-binding transcriptional regulator	Galactose metabolism transcriptional regulator GalR	Change of generic function to specific function	Function described in literature (Zhang et al., 2020)
lp_2514	MFS transporter	Nicotinamide transporter	Change of generic function to specific function	Function described in literature (Kong et al., 2025)
lp_0105	Nickel pincer cofactor biosynthesis protein LarB	Pyridinium-3,5-biscarboxylic acid mononucleotide synthase	Change of generic function to specific function	Function described in literature (Desguin et al., 2016)
lp_0418	Response regulator transcription factor	Plantaricin A-sensing response regulator PlnD	Change of generic function to specific function	Function described in literature (Diep et al., 2001; Diep et al., 2003; Straume et al., 2009)
lp_1814	L-lactate permease	Lactate:proton symporter	Change of name format with additional information	The enzyme is very similar (E-value of 6e-30) to the *E. coli* enzyme, which is known to be a symporter (Núñez et al., 2002)
lp_2370	F0F1 ATP synthase subunit A	F_1_F_o_-ATPase subunit a	Change of name format	The enzyme acts in lactic acid bacteria primarily as an ATP-dependent proton pump (ATPase) rather than an ATP synthase (Koebmann et al., 2000). In addition, the correct format for the subunits of the F_o_ component is lower case letters
lp_0585	Sigma-54-dependent transcriptional regulator	Sigma54 activator protein	Change of function	The annotated name was incorrect, as the protein is not dependent on sigma-54, but rather activates it (Stevens et al., 2010)
lp_2919	Proline iminopeptidase-family hydrolase	Prolinase 2 monomer	Change of name format	Name was changed to match the literature. “monomer” was added to distinguish from the dimeric complex which was created based on the literature (Huang and Tanaka, 2015)
lp_0268	CDP-glycerol glycerophosphotransferase family protein	Teichoic acid poly(glycerol phosphate) polymerase 1	Change of name format	Name modified to correspond to EC name. Serial number added since the organism has two isoforms (Tomita et al., 2013)

## 5 Discussion

The results of this survey suggest that the BioCyc methodology yields a very significant improvement in the quality of curated databases. An average of 25.6% (±8.1% SD) of the proteins encoded in each database had name changes. Our phase I results found that a median of 62% of the assessed changes were functionally informative. Our phase II results then extrapolated these findings to all proteins with name changes, quantifying uncertainty via finite population corrected confidence intervals (Table 2, Column C). When calculated as a percentage of all proteins (data not shown), these values suggested an average of 13% (±5.4% SD) of the proteins within each genome received functionally informative name changes. For organisms encoding many proteins such as *Vibrio cholerae* O1 El Tor N16961 (3,569) and *Synechocystis* sp. PCC 6803 (3,636), this represents a substantial number of informed corrections.

In this analysis, name changes which did not fall under any of our four functional categories reflected stylistic changes that did not modify the proposed enzymatic function. A recent blog, published on 2 June 2026, has found that more than 300 papers, including some published in top journals such as *Nature*, *Nature Medicine*, *Cancer Cell*, *eLife*, and *Science Advance*s, have used the incorrect antibodies in their studies due to a mix-up of protein names, demonstrating the importance of proper protein naming (https://www.science.org/content/article/protein-name-confusion-created-antibody-mix-affecting-hundreds-papers). Therefore, from a wider point of view, we do not consider nomenclature-based name changes as insignificant.

Manual curation of a single BioCyc bacterial genome takes from 2 weeks (example: *Faecalibacterium prausnitzii* A2-165) to 3 person-months (example: *L*. *plantarum* WCFS1). This timeline depends on a variety of factors outside of our control such as the amount of available published literature or pre-existing annotation quality. Despite the significant improvements we observed in the curated databases, we do not claim that curated databases are error-free. First, every genome contains many protein-coding genes that have not been studied experimentally and whose predicted function could be incorrect. Second, although not common, published experimentally supported gene functions can be erroneous. Yet experimental studies are far more reliable than computational studies, and quite often multiple experimental publications, often from multiple labs, confirm the function of a gene product, reducing the risk of error. Even though manual curation itself can introduce errors due to misinterpretation by the curator, a study that evaluated the curation process found a low error rate between 1.4% and 1.58% (Keseler et al., 2014). Ultimately our study quantifies and estimates frequency of functionally informative annotation modifications, but not the accuracy gain resulting from those modifications.

## 6 Conclusion

We have found through a study of 10 microbial genomes that literature-based curation resulted in substantial modifications to protein annotations, with an average of 25.6% (±8.1% SD) protein name changes per PGDB. Our phase I and phase II results suggested that, on average 363, or 13% (±5.4% SD) of the proteins encoded in each genome (approximately half of the name changes per PGDB) received functionally informative changes. Our phase I results found that the rate of such name changes was fairly consistent across genomes, with only two exceptions. Importantly, these changes also influenced the predicted metabolic networks of the organisms, with an average of 89.7 (±32.7) metabolic pathways modified per organism.

The types of data displayed in Tables 1 and 2 underestimates the value of manual curation in our Tier 2 BioCyc databases. BioCyc curators also populate database fields to incorporate data such as enzyme cofactors, kinetic parameters, and subunit structures. Our curation is often accompanied by mini-reviews using appropriate evidence codes and citations linking directly to primary sources of literature, with many Tier 2 databases storing hundreds of citations. Additionally, we also import and store various types of valuable high-throughput data, such as:

- Gene essentiality datasets (under defined conditions)
- Regulatory information (transcription factor binding sites and regulons)
- Expression data from transcriptomic and/or proteomic datasets
- Small regulatory RNA datasets

Expert curation has been shown to reduce the probability of compounding experimental and financial loss (International Society for Biocuration, 2018; de Crécy-Lagard et al., 2022). It is hard to underestimate the importance of annotation corrections of the magnitude reported here. For example, consider a researcher who is analyzing a transcriptomics experiment and has found 10 genes that are upregulated in that experiment. If the genes are chosen independently and the misannotation rate is at least as high as the annotation modification rate (in fact the modification rate should be a lower bound on the misannotation rate because there are bound to be many misannotations that have not been identified in the experimental literature) there is a 77% chance that at least one of those gene functions is misannotated (1-(1-0.135)^10^), thus potentially leading the researcher to mis-interpret the experimental data (0.135 is the average number of proteins per genome receiving functional annotation changes). If a group of 20 genes were analyzed, then the probability that at least one of those genes is misannotated is 94.5%.

The curation practices presented herein elevate the quality of protein annotation in BioCyc’s manually curated databases well beyond that of standard genome annotation pipelines. In addition, they provide original, high-quality syntheses of current knowledge on well-characterized genomic elements and systems, with explicit reference to authoritative and up-to-date primary literature. Curation reduces false functional assignments, clarifies gene functions, and improves pathway completeness.

## Data Availability

The original contributions presented in the study are included in the article, further inquiries can be directed to the corresponding author.

## Footnotes

1 This article contains 3899 words, two tables, and four figures.

## References

[B1] Boneca I. G. de Reuse H. Epinat J. C. Pupin M. Labigne A. Moszer I. (2003). A revised annotation and comparative analysis of *Helicobacter pylori* genomes. Nucleic Acids Res. 31 (6), 1704–1714. 10.1093/nar/gkg250 12626712 PMC152854

[B2] Brenner S. E. (1999). Errors in genome annotation. Trends Genet. 15, 132–133. 10.1016/s0168-9525(99)01706-0 10203816

[B3] Caspi R. Billington R. Keseler I. M. Kothari A. Krummenacker M. Midford P. E. (2020). The MetaCyc database of metabolic pathways and enzymes - a 2019 update. Nucleic Acids Res. 48 (D1), D445–D453. 10.1093/nar/gkz862 31586394 PMC6943030

[B4] de Crécy-Lagard V. Amorin de Hegedus R. Arighi C. Babor J. Bateman A. Blaby I. (2022). A roadmap for the functional annotation of protein families: a community perspective. Database (Oxford) 2022, baac062. 10.1093/database/baac062 35961013 PMC9374478

[B5] Desguin B. Soumillion P. Hols P. Hausinger R. P. (2016). Nickel-pincer cofactor biosynthesis involves LarB-catalyzed pyridinium carboxylation and LarE-dependent sacrificial sulfur insertion. Proc. Natl. Acad. Sci. U. S. A. 113, 5598–5603. 10.1073/pnas.1600486113 27114550 PMC4878509

[B6] Devos D. Valencia A. (2001). Intrinsic errors in genome annotation. Trends Genet. 17, 429–431. 10.1016/s0168-9525(01)02348-4 11485799

[B7] Diep D. B. Johnsborg O. Risøen P. A. Nes I. F. (2001). Evidence for dual functionality of the operon plnABCD in the regulation of bacteriocin production in Lactobacillus plantarum. Mol. Microbiol. 41, 633–644. 10.1046/j.1365-2958.2001.02533.x 11532131

[B8] Diep D. B. Myhre R. Johnsborg O. Aakra A. Nes I. F. (2003). Inducible bacteriocin production in lactobacillus is regulated by differential expression of the pln operons and by two antagonizing response regulators, the activity of which is enhanced upon phosphorylation. Mol. Microbiol. 47, 483–494. 10.1046/j.1365-2958.2003.03310.x 12519198

[B9] Esteban-Torres M. Reverón I. Mancheño J. M. de Las Rivas B. Muñoz R. (2013). Characterization of a feruloyl esterase from Lactobacillus plantarum. Appl. Environ. Microbiol. 79, 5130–5136. 10.1128/AEM.01523-13 23793626 PMC3753946

[B10] Gross G. Snel J. Boekhorst J. Smits M. A. Kleerebezem M. (2010). Biodiversity of mannose-specific adhesion in Lactobacillus plantarum revisited: strain-specific domain composition of the mannose-adhesin. Benef. Microbes 1, 61–66. 10.3920/BM2008.1006 21840797

[B11] Howe D. Costanzo M. Fey P. Gojobori T. Hannick L. Hide W. (2008). Big data: the future of biocuration. Nature 455, 47–50. 10.1038/455047a 18769432 PMC2819144

[B12] Huang Y. Tanaka T. (2015). Characterization of two putative prolinases (PepR1 and PepR2) from Lactobacillus plantarum WCFS1: occurrence of two isozymes with structural similarity and different catalytic properties. Biochim. Biophys. Acta 1854, 91–100. 10.1016/j.bbapap.2014.11.003 25463046

[B13] International Society for Biocuration (2018). Biocuration: distilling data into knowledge. PLoS Biol. 16, e2002846. 10.1371/journal.pbio.2002846 29659566 PMC5919672

[B14] Janssen A. B. Gibson P. S. Bravo A. M. de Bakker V. Slager J. Veening J. W. (2025). PneumoBrowse 2: an integrated visual platform for curated genome annotation and multiomics data analysis of Streptococcus pneumoniae. Nucleic Acids Res. 53 (D1), D839–D851. 10.1093/nar/gkae923 39436044 PMC11701578

[B15] Jiang Y. Oron T. R. Clark W. T. Bankapur A. R. D’Andrea D. Lepore R. (2016). An expanded evaluation of protein function prediction methods shows an improvement in accuracy. Genome Biol. 17, 184. 10.1186/s13059-016-1037-6 27604469 PMC5015320

[B16] Junker V. L. Apweiler R. Bairoch A. (1999). Representation of functional information in the SWISS-PROT data bank. Bioinformatics 15, 1066–1067. 10.1093/bioinformatics/15.12.1066 10746001

[B17] Keseler I. M. Skrzypek M. Weerasinghe D. Chen A. Y. Fulcher C. Li G.-W. (2014). Curation accuracy of model organism databases. Database (Oxford) 2014, bau058. 10.1093/database/bau058 24923819 PMC4207230

[B18] Koebmann B. J. Nilsson D. Kuipers O. P. Jensen P. R. (2000). The membrane-bound H(+)-ATPase complex is essential for growth of Lactococcus lactis. J. Bacteriol. 182, 4738–4743. 10.1128/JB.182.17.4738-4743.2000 10940012 PMC111348

[B19] Kong L. Li X. He Q. Yao Q. Ai L. Qin J. (2025). Transcriptome-guided engineering of a native niacin transporter in Lactiplantibacillus plantarum unveils metabolic rewiring for NMN biosynthesis. Front. Microbiol. 16, 1637666. 10.3389/fmicb.2025.1637666 40727558 PMC12301409

[B20] Navarro-González I. Alvaro S.-F. Francisco G.-C. (2013). Overexpression, purification, and biochemical characterization of the esterase Est0796 from Lactobacillus plantarum WCFS1. Mol. Biotechnol. 54, 651–660. 10.1007/s12033-012-9607-7 23054632

[B21] Novichkov P. S. Kazakov A. E. Ravcheev D. A. Leyn S. A. Kovaleva G. Y. Sutormin R. A. (2013). RegPrecise 3.0--a resource for genome-scale exploration of transcriptional regulation in bacteria. BMC Genomics 14, 745. 10.1186/1471-2164-14-745 24175918 PMC3840689

[B22] Núñez M. F. Kwon O. Wilson T. H. Aguilar J. Baldoma L. Lin E. C. C. (2002). Transport of L-Lactate, D-Lactate, and glycolate by the LldP and GlcA membrane carriers of *Escherichia coli* . Biochem. Biophys. Res. Commun. 290, 824–829. 10.1006/bbrc.2001.6255 11785976

[B23] Percudani R. Carnevali D. Puggioni V. (2013). Ureidoglycolate hydrolase, amidohydrolase, lyase: how errors in biological databases are incorporated in scientific papers and *vice versa* . Database (Oxford) 2013, bat071. 10.1093/database/bat071 24107613 PMC3793230

[B24] Pretzer G. Snel J. Molenaar D. Wiersma A. Bron P. A. Lambert J. (2005). Biodiversity-based identification and functional characterization of the mannose-specific adhesin of Lactobacillus plantarum. J. Bacteriol. 187, 6128–6136. 10.1128/JB.187.17.6128-6136.2005 16109954 PMC1196140

[B25] Radivojac P. Clark W. T. Oron T. R. Schnoes A. M. Wittkop T. Sokolov A. (2013). A large-scale evaluation of computational protein function prediction. Nat. Methods 10, 221–227. 10.1038/nmeth.2340 23353650 PMC3584181

[B26] Remus D. M. van Kranenburg R. van Swam I. I. Taverne N. Bongers R. S. Wels M. (2012). Impact of 4 Lactobacillus plantarum capsular polysaccharide clusters on surface glycan composition and host cell signaling. Microb. Cell Fact. 11, 149. 10.1186/1475-2859-11-149 23170998 PMC3539956

[B27] Saulnier D. M. A. Molenaar D. de Vos W. M. Gibson G. R. Kolida S. (2007). Identification of prebiotic fructooligosaccharide metabolism in Lactobacillus plantarum WCFS1 through microarrays. Appl. Environ. Microbiol. 73, 1753–1765. 10.1128/AEM.01151-06 17261521 PMC1828832

[B28] Schwengers O. Jelonek L. Dieckmann M. A. Beyvers S. Blom J. Goesmann A. (2021). Bakta: rapid and standardized annotation of bacterial genomes *via* alignment-free sequence identification. Microb. Genom 7, 000685. 10.1099/mgen.0.000685 34739369 PMC8743544

[B29] Seemann T. (2014). Prokka: rapid prokaryotic genome annotation. Bioinformatics 30, 2068–2069. 10.1093/bioinformatics/btu153 24642063

[B30] Slager J. Aprianto R. Veening J. W. (2018). Deep genome annotation of the opportunistic human pathogen Streptococcus pneumoniae D39. Nucleic Acids Res. 46 (19), 9971–9989. 10.1093/nar/gky725 30107613 PMC6212727

[B31] Stevens M. J. A. Molenaar D. de Jong A. De Vos W. M. Kleerebezem M. (2010). sigma54-Mediated control of the mannose phosphotransferase sytem in Lactobacillus plantarum impacts on carbohydrate metabolism. Microbiol. Read. 156, 695–707. 10.1099/mic.0.034165-0 19942662

[B32] Straume D. Johansen R. F. Bjørås M. Nes I. F. Diep D. B. (2009). DNA binding kinetics of two response regulators, PlnC and PlnD, from the bacteriocin regulon of Lactobacillus plantarum C11. BMC Biochem. 10, 17. 10.1186/1471-2091-10-17 19519894 PMC2714321

[B33] Tatusova T. DiCuccio M. Badretdin A. Chetvernin V. Nawrocki E. P. Zaslavsky L. (2016). NCBI prokaryotic genome annotation pipeline. Nucleic Acids Res. 44, 6614–6624. 10.1093/nar/gkw569 27342282 PMC5001611

[B34] Tomb J. F. White O. Kerlavage A. R. Clayton R. A. Sutton G. G. Fleischmann R. D. (1997). The complete genome sequence of the gastric pathogen *Helicobacter pylori* . Nature 388 (6642), 539–547. 10.1038/41483 9252185

[B35] Tomita S. de Waard P. Bakx E. J. Schols H. A. Kleerebezem M. Bron P. A. (2013). The structure of an alternative wall teichoic acid produced by a Lactobacillus plantarum WCFS1 mutant contains a 1,5-linked poly(ribitol phosphate) backbone with 2-α-D-glucosyl substitutions. Carbohydr. Res. 370, 67–71. 10.1016/j.carres.2013.01.018 23454138

[B36] UniProt Consortium (2025). UniProt: the universal protein knowledgebase in 2025. Nucleic Acids Res. 53 (D1), D609–D617. 10.1093/nar/gkae1010 39552041 PMC11701636

[B37] Zhang S. S. Xu Z. S. Qin L. H. Kong J. (2020). Low-sugar yogurt making by the co-cultivation of Lactobacillus plantarum WCFS1 with yogurt starter cultures. J. Dairy Sci. 103, 3045–3054. 10.3168/jds.2019-17347 32059863

